# Decrease of survivin, p53 and Bcl-2 expression in chemorefractory colorectal liver metastases may be predictive of radiosensivity after radioembolization with yttrium-90 resin microspheres

**DOI:** 10.1186/1756-9966-32-13

**Published:** 2013-03-06

**Authors:** Elisa Melucci, Maurizio Cosimelli, Livio Carpanese, Giuseppe Pizzi, Francesco Izzo, Francesco Fiore, Rita Golfieri, Emanuela Giampalma, Isabella Sperduti, Cristiana Ercolani, Rosa Sciuto, Raffaello Mancini, Carlo Garufi, Maria Grazia Diodoro, Marcella Mottolese

**Affiliations:** 1 Department of Pathology, Regina Elena National Cancer Institute, Rome, Italy; 2 Department of Surgery, Regina Elena National Cancer Institute, Rome, Italy; 3 Department of Interventional Radiology, Regina Elena National Cancer Institute, Rome, Italy; 4 Department of Surgery, Pascale Cancer Institute, Naples, Italy; 5 Department of Interventional Radiology, Pascale Cancer Institute, Naples, Italy; 6 Department of Interventional Radiology, Malpighi Hospital, Bologna, Italy; 7 Biostatistics, Scientific Direction, Regina Elena National Cancer Institute, Rome, Italy; 8 Department of Nuclear Medicine, Regina Elena National Cancer Institute, Rome, Italy; 9 Medical oncology, Regina Elena National Cancer Institute, Rome, Italy

**Keywords:** Colorectal cancer, Liver metastases, Radioembolization, Yttrium-90-resin microspheres, Survivin, p53, Bcl-2, Ki-67

## Abstract

In a prospective multicenter phase II trial of radioembolization with yttrium-90 (^90^Y-RE) in chemorefractory liver-dominant metastatic colorectal cancer (mCRC), we showed that median survival was 12.6 months (95% CI 7.0–18.3) with 48% of 50 patients achieving disease control. In this extension retrospective study, we analyzed whether a panel of biomarkers, known to be associated to an adverse clinical outcome, underwent variations in CRC liver metastases pre and post ^90^Y-RE.

Of the 50 patients included in the study, 29 pre-^90^Y-RE therapy and 15 post-^90^Y-RE had liver biopsy specimens available. In these series we investigated survivin, p53, Bcl-2 and Ki-67 expression pre- and post-^90^Y-RE by immuhistochemistry (IHC). Our findings evidenced a decrease of survivin (77% vs 33%), p53 (93% vs 73%), Bcl-2 (37% vs 26%) expression as well as of Ki-67 proliferation index (62.5% vs 40%) on liver biopsies collected post-^90^Y-RE as compared to pre-^90^Y-RE. In the subset of 13 matched liver metastases we further confirmed the reduction of survivin (92.3% vs 53.8%; p = 0.06), p53 (100% vs 69.2%; p = 0.05) and Bcl-2 (69.2% vs 53.8%; p = 0.05) expression post-^90^Y-RE. This biomarker modulation was accompanied by morphological changes as steatohepatitis, hepatocyte necrosis, collagen deposition, proliferating and/or bile duct ectasia, focal sinusoidal dilatation and fibrosis.

Although our analysis was conducted in a very limited number cases, these changes appear strictly related to the response to ^90^Y-RE therapy and may deserve further investigation on a larger series of patients.

## Introduction

Liver metastases are a significant cause of morbidity and mortality for more than 45% of patients who present with colorectal cancer (CRC) [1]. Although chemotherapy regimens combined with biologic agents have improved the control of liver metastases, the occurrence of hepatic metastases continues to present a life-limiting prognosis for most patients with advanced CRC [2] being 5 year survival approximately 11%. In the setting of clinical trials, median overall survival for unresectable metastases have been extended beyond two years using combinations including oxaliplatin, irinotecan, capecitabine and biologic agents (bevacizumab, cetuximab, panitumumab) [3,4]. In parallel with these developments, the application of locally ablative procedures, such as radiofrequency ablation, are increasingly considered beneficial for patients with unresectable liver-only disease who present with tumors ≤ 3–4 cm in diameter. These regional treatments for liver metastases can also be used to consolidate the treatment response with chemotherapy, in order to further increase the number of patients eligible for resection [5,6]. Despite these gains, one of the major challenges in advanced CRC are the growing proportion of patients who continue to present with progressive liver involvement having exhausted all other therapeutic options.

Radioembolization with yttrium-90 (^90^Y-RE) and, as recently described, with holmium-166 poly (L-lactic acid) labeled microspheres (^166^Ho-PLLA-MS) [7], are therapeutic procedures applied to the liver that allow direct delivery of high-dose radiation to liver tumors (both primary and metastatic) by means of endovascular catheters, selectively placed within the hepatic arterial vasculature. ^90^Y and ^166^Ho-PLLA-MS (resin or glass) microspheres lodge within the neovascular rim of the tumor(s) [8,9].

In a multicenter phase II trial conducted in highly chemorefractory liver-dominant metastatic CRC (mCRC), we showed that 48% (24 of 50) of patients achieved disease control with a median overall survival of 12.6 months following RE with ^90^Y-radiolabelled resin microspheres [10]. This finding is consistent with the results from other multicenter evaluations using ^90^Y-RE in the chemorefractory setting [11]. Up to date, there are no studies which have investigated biomarker expression and response to ^90^Y-RE therapy.

It is largely described that the ability to avoid apoptosis is one of the major oncogenic switches contributing to tumor progression. Among the gene coding apoptosis and cell proliferation protein regulators, Bcl-2, an antiapopototic protein, survivin, one of the member of the inhibitor of apoptosis (IAP) protein family and p53 may identify CRC patients at a higher risk of tumor progression [12-14].

In the present retrospective study which is an extension of our previous one [10], we evaluated whether the expression of these biomarkers may undergo to significant changes before and after ^90^Y-RE thus providing predictive information of clinical value.

## Methods

### Patients and treatment

Between May 2005 and August 2007, 50 patients with unresectable, histologically proven CRC liver metastases and limited extra-hepatic disease (≤ 3 nodules in the same extra-hepatic organ each < 3 mm), in progression following standard systemic chemotherapy, were recruited from four Italian centers in a phase II prospective clinical trial conducted by the Italian Society of Locoregional Therapy in Oncology (SITILO). Further details of the treatment planning and patient selection have been outlined in our previous paper [10]. In brief, patients were required to be between 18 and 75 years of age, have liver metastases measurable by Response Evaluation Criteria in Solid Tumours (RECIST), adequate renal function (creatinine < 1.5 7 × normal values or creatinine clearance > 50 mL/minute), hemopoietic function, WHO or ECOG performance status ≤ 2 and were able to give informed consent. To be eligible for ^90^Y-RE, patients were required to have: sufficient liver function; hepatic arterial anatomy that would enable safe delivery of microspheres to the liver only; liver to lung shunting of < 20% on a pre-treatment technetium-99m labeled macro-aggregated-albumin (^99m^Tc-MAA) nuclear scan; and a patent main portal vein. Patients were excluded if they were pregnant, had evidence of local recurrence of primary disease, inflammatory gastrointestinal disease or had received prior treatment with hepatic arterial chemotherapy or external beam radiotherapy to the liver. The median interval between diagnosis of mCRC and ^90^Y-RE was 17 months (range, 6–71 months). To investigate biomarkers expression and response to ^90^Y-RE therapy, liver metastases biopsies were taken 8–21 days prior to ^90^Y-RE and 2 months post-^90^Y-RE. Tissue specimens were available from 29 patients pre therapy and 15 patients post therapy. Samples pre- and post-^90^Y-RE were concomitantly available in 13 patients.

The study was approved by the Ethical Committee at the Regina Elena Cancer Institute (N°534; 22/03/05) and a written informed consent was obtained by all patients.

### Immunohistochemistry

Formalin-fixed paraffin-embedded liver biopsies were cut on SuperFrost Plus slides (Menzel-Gläser, Braunschweig, Germany). Antigen retrieval was performed at 96°C (10 mM/L citrate buffer, pH 6) for 40 minutes in a thermostatic bath. Sections were incubated with the polyclonal antibody (PAb) anti-survivin (1:100, Novus Biological, DBA, Milan, Italy); with the anti-Ki-67 monoclonal antibody (MoAb) MIB-1 (5 μg/ml; Dako, Milan, Italy), the anti-p53 MoAb DO7 (5 μg/ml, Dako), the anti-Bcl-2 MoAb 124 (1,5 μg/ml; Dako) for 30 minutes at room temperature. Positive and negative controls were included for each antibody and in each batch of staining. Immunoreactions were revealed by a streptavidin-biotin enhanced immunoperoxidase technique (Super Sensitive MultiLink Menarini, Florence, Italy) in an automated autostainer. Diaminobenzidine was used as chromogenic substrate.

Results were considered positive for survivin when at least 20% of tumor cells, independent of nuclear or cytoplasmic localization, were immunostained, for p53 when 10% of tumor cell nuclei were labelled, for Bcl-2 when > 5% of cells showed a cytoplasmic immunoreaction. Ki-67 proliferation index, based on the median value of our series, was regarded as high if greater than 50% of the cell nuclei were immunostained. Only well preserved tumor areas were considered for IHC evaluation. The IHC results were evaluated independently and in a blinded manner by two investigators (MD, MM).

### Statistical analysis

The correlation between biomarkers expression and the response to ^90^Y-RE was tested by the Pearson Chi-Square test and Mac Nemar test. Significance was assessed at 5% level (*p* < 0.05). The SPSS statistical software package version 19.0 was used for analyses (SPSS, Inc, Chicago, IL, USA).

## Results

### Expression pattern of survivin, p53, Bcl-2 and Ki-67 in liver metastases pre- and post-^90^Y-RE

Of the 50 patients included in the SITILO clinical trial, 29 pre-^90^Y-RE and 15 post-^90^Y-RE had sufficient tissue material from their liver metastases for IHC evaluation of survivin, p53, Bcl-2 and Ki-67. As reported in Table 1, we found that, of the 29 liver metastases analyzed pre-^90^Y-RE, 24 (77.4%) were survivin positive, 27 (93.1%) p53 positive,11 (37.9%) Bcl-2 positive and 18 (62.5%) presented a high Ki-67 proliferation index (>50%). Of the 15 liver metastases available post-^90^Y-RE, survivin was expressed in 5 cases (33.3%), p53 in 11 (73.3%), Bcl-2 in 4 (26.7%) and Ki-67 was high in 6 lesions (40.0%) evidencing a variation in biomarker expression pre and post-^90^Y-RE.

**Table 1:** **Expression pattern of survivin, p53 and Bcl-2 in liver metastases pre- and post -**^**90**^**Y-RE**

**Biomarkers**	**Number of patients (%)**	
	**Pre-**^**90**^**Y-RE (N = 29)**	**Post-**^**90**^**Y-RE (N = 15)**
**Survivin N (%)**		
Negative (≤ 20)	5 (16.2)	10 (66.7)
Positive (> 20)	24 (77.4)	5 (33.3)
**p53 N (%)**		
Negative (≤ 10)	2 (6.9)	4 (26.7)
Positive (> 10)	27 (93.1)	11 (73.3)
**Bcl-2 N (%)**		
Negative (≤ 5)	18 (62.1)	11 (73.3)
Positive (> 5)	11 (37.9)	4 (26.7)
**Ki-67 N (%)**		
Negative (<50)	11 (37.5)	9 (60.0)
Positive (≥ 50)	18 (62.5)	6 (40.0)

### Changes of survivin, p53, Bcl-2 and Ki-67 in the 13 matched liver metastases pre- and post-^90^Y-RE

In our series of liver biopsies, 13 patients had matched valuable tissues pre and post-^90^Y-RE. As reported in Table 2, the 13 paired patients, included in biomarker analysis, were found to be representative of the overall cohort of the 50 patients enrolled in the SITILO clinical trial with no statistical differences between the groups for baseline parameters (sex, site of primary tumors, number of metastases, liver involvement, performance status, bevacizumab or cetuximab therapy). On the basis of this comparative analysis, we evaluated whether survivin, p53, Bcl-2 and Ki-67 expression varied pre- and post-^90^Y-RE therapy in our series of 13 matched patients.

**Table 2:** **Comparison of clinical variables between the overall series of patients and the series with liver biopsies pre- and post-**^**90**^**Y-RE**

**Baseline Characteristics**																	
**Patients**	Age (years)*	Time to RE**	FU months***	**Sex N° (%)**		**PT site N° (%)**		**Met N° (%)**		**Liver involvement N° (%)**		**PS N° (%)**		**Pre BV N° (%)**		**Pre CTX N° (%)**	
				**M**	**F**	**Colon**	**Rectum**	**≤ 4**	**> 4**	**<25%**	**> 25%**	**0**	**≥ 1**	**No**	**Yes**	**No**	**Yes**
**Overall Series (N = 50)**	64	19	14	37	13	41	9	21	29	20	7	35	15	39	11	45	5
	(34–38)	(6–71)	(2–49)	(74)	(26)	(82)	(18)	(42)	(58)	(40)	(54)	(70)	(30)	(78)	(22)	(90)	(10)
**Pre/Post RE series (N = 13)**	58	21	15	9	4	11	2	4	9	30	6	9	4	9	4	12	1
	(40–75)	(9–53)	(3–49)	(69)	(31)	(85)	(15)	(31)	(69)	(60)	(46)	(69)	(31)	(69)	(31)	(92)	(8)
**P value**	**0.11**	**0.50**	**0.99**	**0.49**		**0.99**		**0.54**		**0.54**		**0.99**		**0.49**		**0.99**	

* mean (range); ** Months from diagnosis to ^90^Y-RE; ***Follow up post-^90^Y-RE;

*M*, male; *F*, female; *PT*, Primary Tumor; *Met*, Metastases; *PS*, Performance Status; *BV*, bevacizumab; *CTX*, cetuximab.

**Figure 1:**
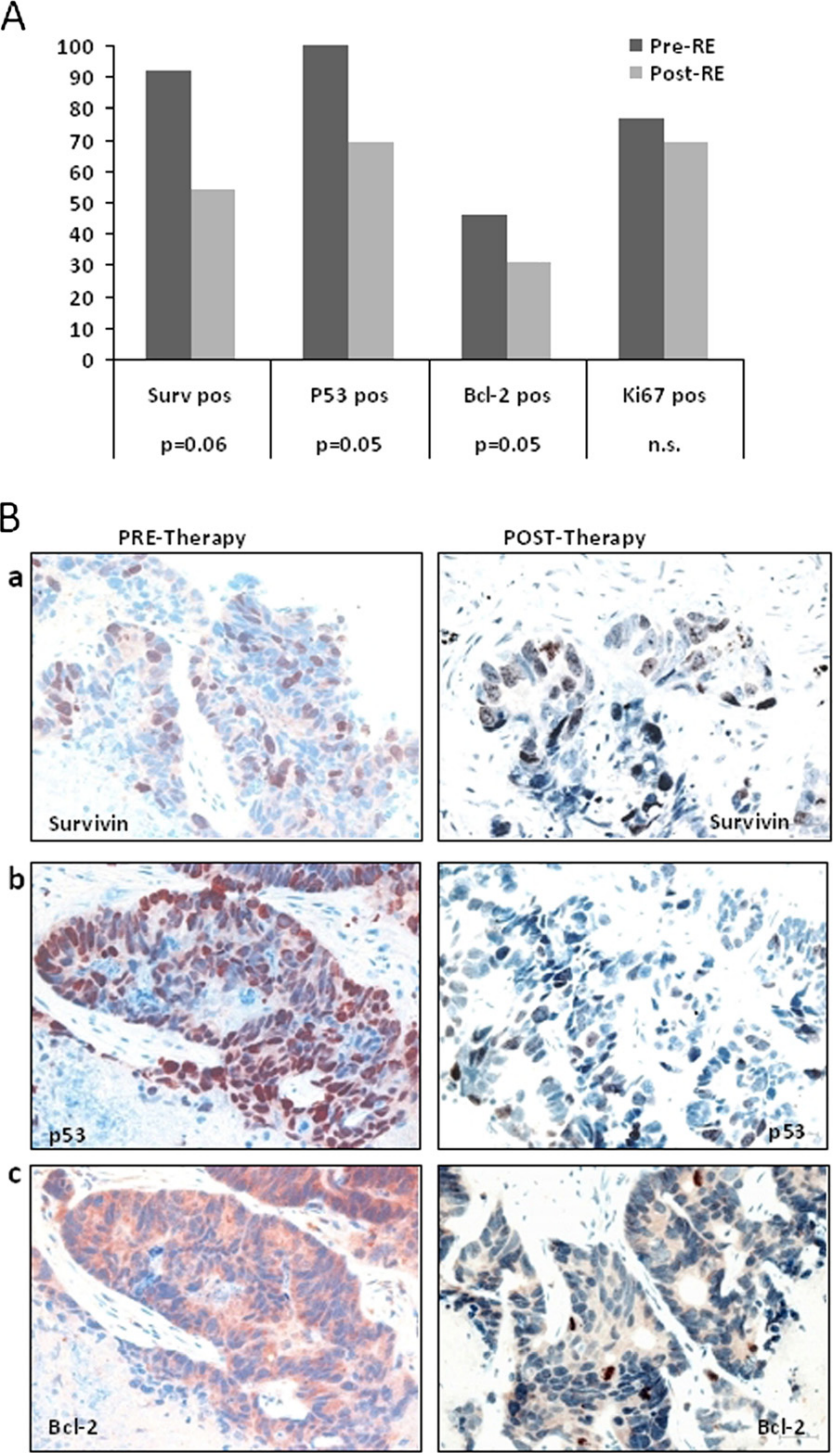
**Changes of survivin, p53, Bcl-2 and Ki-67 in liver metastases pre- and post-**^**90**^**Y-RE. A**. The histogram shows the significant reduction of the positivity of survivin (from 92% to 54%; *p =* 0.06), p53 (from 100% to 69%; *p* = 0.05) and Bcl-2 (from 46% to 31%; *p* = 0.05) expression in liver metastases pre- and post-^90^Y-RE therapy. A limited, not significant decrease in proliferation index by Ki-67 (from 77% to 61%) is also evident. **B**. Immunohistochemical staining of 3 autologous liver metastases sampled pre- and post- therapy showing a strong decrease in survivin (**a**) p53 (**b**), and Bcl-2 (**c**) immunoreactions.

As described in Figure 1 panel A, the IHC biomarker analysis in this subset of mCRC showed that post-^90^Y-RE there was a significant reduction in survivin positivity (from 92% to 54% of samples; p *=* 0.06) and p53 nuclear accumulation (from 100% to 69%; p = 0.05) (Figure 1 panel B-a and B-b). Furthermore, we found a small, but significant, decrease in Bcl-2 positivity (from 46% to 31%; *p* = 0.05; Figure 1 panel B-c) and a limited, not significant, decrease in Ki-67 positivity (from 77% to 61%).

**Figure 2:**
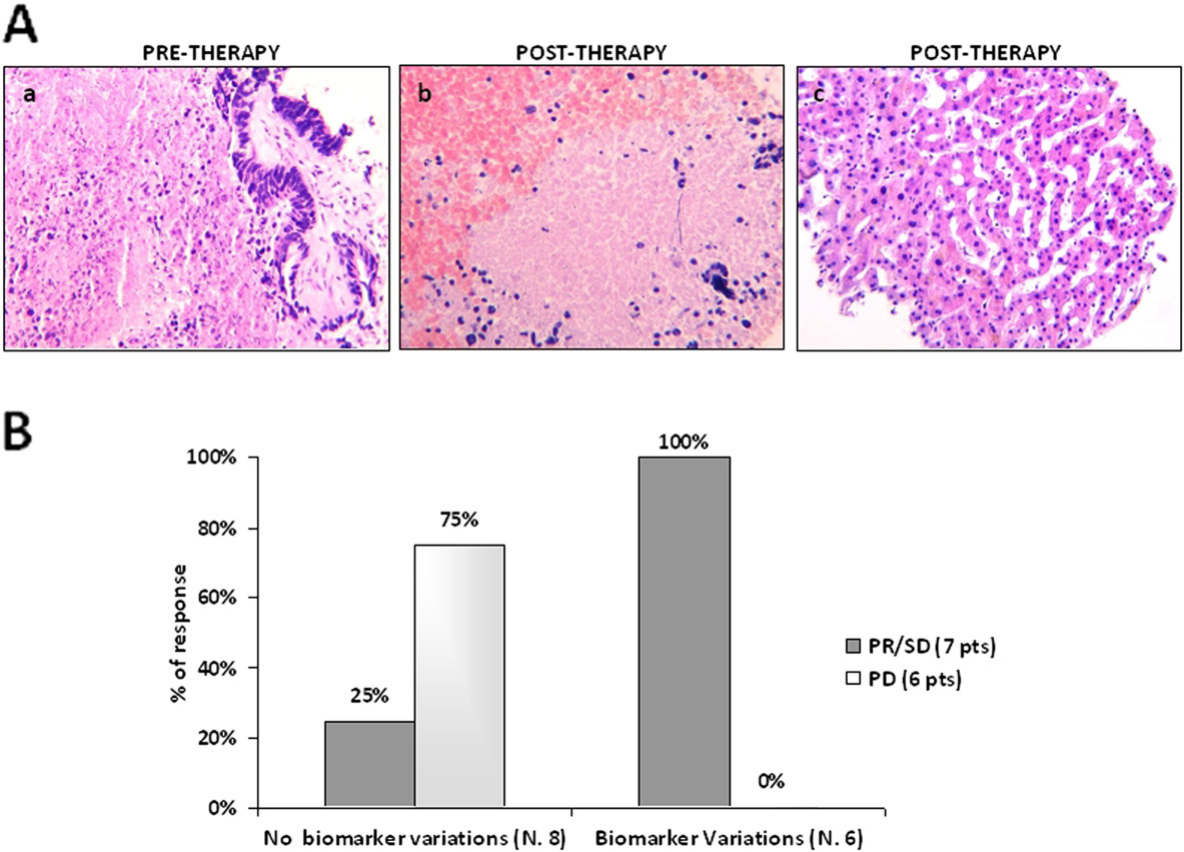
**Morphological and phenotypic changes in paired liver metastases pre- and post- **^**90**^**Y-RE. A**. Example of histological features in a pre-^90^Y-RE CRC liver metastasis with focal areas of necrosis (**a**), in a post-^90^Y-RE CRC liver metastasis with evident increase of tumor necrosis (**b**) and, within uninvolved peritumoral liver parenchyma, showing dysplastic hepatocytes, sinusoidal dilatation, leukocyte infiltration and bile-duct proliferation (**c**). **B**. Histogram summarizing Sirtex response in the 13 autologous liver biopsies according to biomarker changes pre- and post- therapy. Two patients (25%) not showing biomarker changes suffered PD whereas 6 patients (100%) showing biomarker changes had PR or SD.

Concerning histological features, we observed that liver metastases sampled post-^90^Y-RE presented more abundant necrosis, with only occasional residual cancer cells, than those sampled pre-^90^Y-RE (Figure 2, panel A-a, A-b). The adjacent liver parenchyma, in both pre- and post-treatment samples, showed evidence of tissue damage from prior chemotherapy including: steatohepatitis, hepatocyte necrosis, collagen deposition, proliferating and/or bile duct ectasia, focal sinusoidal dilatation and fibrosis (Figure 2, panel A-c).

### Biomarker variation and response rate pre and post-^90^Y-RE in 13 paired liver metastases

In our series of 13 matched patients, 5 presented biomarker variations pre and post-^90^Y-RE therapy and 8 no biomarker variations. Of clinical interest, 6 of the latter patients (75%) presented progression disease whereas all the 5 patients showing changes in biomarker expression had partial response or stable disease (Figure 2, panel B). Nevertheless, the limited number of patients did not allow us to determine whether these changes may really affect survival.

## Discussion

Patients included in the present study were from a multicenter phase II clinical trial which is the first prospective evaluation of ^90^Y-RE in CRC patients with liver metastases who failed previous oxaliplatinum and irinotecan based chemotherapy regimen [10]. It has been widely reported that alterations in genes, as survivin, p53 and Bcl-2, which regulate cell growth and apoptotic processes, are significantly associated to an unfavourable clinical outcome in CRC patients [15]. In our series of 29 liver mCRC patients, we found that most tumors sampled prior to ^90^Y-RE were p53, survivin, and Bcl-2 highly positive and presented a high Ki-67 proliferation index. In contrast, we found a significant reduction in p53, survivin and Bcl-2 positive expression in liver metastasis sampled two months post-^90^Y-RE. There was also a trend towards a reduction in cells with a high proliferative index as measured by Ki-67. We have previously shown that colon cancers harboring p53 nuclear accumulation, as assessed by the DO7 anti-p53 antibody, represent a subset of tumors with a more aggressive clinical behaviour in patients with stage II tumors as well as in young patients [13,16]. Furthermore, several studies have shown an increased incidence of p53 nuclear accumulation in liver metastases in comparison to the primary tumor, hypothesizing a role for p53 in CRC liver metastatization. In particular, the presence of ≥ 3 liver metastases identified a subset of patients with a very poor prognosis mainly when associated to p53 mutations [17]. A number of studies have also shown that tumors that do not express detectable levels of Bcl-2, but which exhibited nuclear accumulation of p53, were associated with the shortest patient survival, while Bcl-2-positive and p53-negative tumors had the best prognosis [12,17]. Studies conducted at our Institute showed that p53 positivity combined with Bcl-2 negativity and elevated Ki-67 score correlated with advanced tumor stage, poorly differentiated tumors and increased probability of relapse. Also elevated survivin expression levels in primary CRC are related to decreased survival [14,15]. In resected liver tumors, altered expression of survivin, p53, Ki-67 and, more recently, KRAS mutations, have been shown to be independently predictive of hepatic recurrence and poor survival [13,16,18]. It is recently reported that defective mismatch repair predicts resistance to 5-fluorouracil (5FU) and KRAS mutation resistance to anti-EGFR antibody therapy [19]. Nevertheless, no predictive markers of RE efficacy in mCRC have been identified up to now. In terms of the predictive response to radiotherapy, several studies have linked epidermal growth factor receptor (EGFR) and vascular endothelial growth factor (VEGF) expression to a lack of response to pre-operative radiotherapy in locally advanced rectal cancer [19-21]. Neither p53, Ki-67 and survivin expression appear to be correlated to pre-operative chemo-radiotherapy response and prognosis in locally advanced rectal cancer [22,23]. To date, however, no study has evaluated the predictive value of molecular markers on radiosensitivity of CRC liver metastasis. In this context, our findings, although in a very limited number of patients, may be clinically relevant.

The rapid changes of biomarkers observed in our series post-^90^Y-RE may be due to clonal selection or to epigenetic changes, not previously recorded in this context. Such mechanisms are usually discussed in the context of cell adaption to chemotherapy and evolving resistance. Radio-sensitivity of colorectal cancer cells may be determined by p53 mutation [23,24], whereas there is no evidence that chemotherapy per se cause changes in the cellular expression of p53 [25]. This is the first time that we have recorded a down-staging in p53 protein expression after ^90^Y-RE.

It is likely that both disease progression and a prolonged prior chemotherapy affected the efficacy and tolerability of ^90^Y-RE in the liver. In fact, mild manifestations of non-alcoholic fatty liver disease (NAFLD) after 5FU [26], more serious non-alcoholic steatohepatitis after irinotecan and sinusoidal obstruction syndrome (SOS) after oxaliplatin-based treatment [27] have been recorded. Using the same biomarkers as in our study, Panasiuk and colleagues [28] showed that the intensification of inflammation in NAFLD may also impact on biomarker expression in human hepatocytes with the induction of pro-apoptotic protein p53 and the inhibition of anti-apoptotic Bcl-2.

There are clear limitations to our study, not least of which was the small patient numbers and limited tissue sampling. Nevertheless, we believe that our findings merit further investigation in prospective clinical trials. We are planning to evaluate this biomarker panel in a phase II randomized trial on 2nd line treatment. KRAS mutated CRC patients with unresectable liver metastasis will be randomized to receive systemic therapy vs systemic therapy plus ^90^Y-RE. The combined assessment of survivin, p53 and Bcl-2 pre and post-^90^Y-RE therapy may improve our ability to predict outcomes in the treatment paradigm of metastatic KRAS mutated CRC patients.

## Competing interests

The authors declared that they have no competing interest.

## Authors’ contributions

EM and CE carried out immunohistochemical staining and contributed in data acquirement and interpretation. MC contributed to the study design, data interpretation and manuscript drafting. LC, GP, FF, RG, EG performed liver biopsies pre and post radioembolization in all the patients included in this study. IS was responsible for the database set up and for the statistical analyses. RS was involved in the patient treatment with ytttium-90 microspheres. MD evaluated the morphological features of liver biopsies and revised all the slides submitted to immunohistochemical staining. CG and FI, RM provided clinical and surgical data of the patients including treatment schedule and follow up. MM were responsible for the study concept and design and for the interpretation of results, helped in data discussion, critically revised the manuscript for important intellectual content, and obtained funding for the study. All authors have read and approved the manuscript.
